# Optimization of the Ultrasonic-Assisted Extraction of Phenolic Compounds from *Oryza Sativa* L. ‘Violet Nori’ and Determination of the Antioxidant Properties of its Caryopses and Leaves

**DOI:** 10.3390/molecules23040844

**Published:** 2018-04-07

**Authors:** Federica Turrini, Raffaella Boggia, Riccardo Leardi, Matilde Borriello, Paola Zunin

**Affiliations:** 1 Department of Pharmacy, University of Genova, 16148, Genova, Italy; turrini@difar.unige.it (F.T.); boggia@difar.unige.it (R.B.); riclea@difar.unige.it (R.L.); 2 Marco Polo Institute, Via Angelo Sciaccaluga 9, 16147, Genova, Italy; matiborriello59@gmail.com

**Keywords:** Ultrasound-Assisted Extraction (UAE), antioxidant activity, anthocyanins, violet rice, violet leaves

## Abstract

‘Violet Nori’ is a new spontaneous growing violet rice variety showing a peculiar violet color in its fresh leaves as well. In this paper, the antioxidant properties and the content of total phenols, radical scavengers, and anthocyanins in ‘Violet Nori’ caryopses, flour, and leaves are explored and compared. Ultrasonic-Assisted Extraction (UAE) is employed for the extraction of phenolic compounds, improving the extraction conditions by Design of Experiments (DoE). The obtained results show that the Radical Scavenging Activities (RSAs), the Total Phenolic Contents (TPCs), and the anthocyanin amounts (1000–1500 μg/g, expressed as cyanidin-3-glucoside) of ‘Violet Nori’ caryopses are higher than those in the other analyzed colored rice samples (300–900 μg/g as cyanidin-3-glucoside), with the exception of the cultivars ‘Artemide’ and ‘Nerone’, which show comparable values of RSAs and TPCs. The study of ‘Violet Nori’ leaves at different plant maturation stages shows that their anthocyanin content is 2–3 times higher than in the caryopses and in the flour, reaching the highest levels at about 60 days from seeding. Thus, the estimated extraction yield of 4 kg anthocyanins/t makes fresh leaves very interesting for the extraction of anthocyanins on an industrial scale, whereas violet caryopses are a very interesting dietetic source of valuable anthocyanins and other antioxidant compounds.

## 1. Introduction

The interest in the healthy properties of colored rice (*Oryza sativa* L.) is expanding all over the world, and the consumption of black and red rice is increasing every year. In Italy, for example, in the last six years colored rice crop areas have increased by about 700% [1]. Several authors reported that anthocyanins, which accumulate in the rice caryopsis during maturation, are the major identified pigments in black and red rice [2,3,4]. These molecules have functional properties mainly related to their antioxidant activity [5,6,7,8,9], but also other health promoting activities, such as anti-carcinogenic [10,11] and anti-inflammatory properties [12], have been reported. Opposingly, in 2012 Chen [13] reported that the antioxidant activity of colored rice is rather related to the other phenolic compounds of rice, such as flavonols and flavan-3-ols, which contribute to health-promoting activities by radical scavenging and by their protection against cardiovascular diseases.

*Oryza sativa* L. ‘Violet Nori’ is a new violet, aromatic rice spontaneously grown in Piedmont (Italy) and registered at the Community Plant Variety Office (CPVO). The distinguishing feature of its plant is that both its fresh leaves and its caryopses have an intense violet color, which triggers the interest of food industry in violet leaves as cheap raw material for the industrial extraction of anthocyanins suitable to be employed as natural food colorants and for the preparation of enriched foods. As far as violet caryopses are concerned, they stand out for their intense and pleasant aromatic flavor, for their size and consistency, for their cooking resistance, and for their peculiar violet color, which leads to the assumption of a high content of anthocyanins. 

Since rice can be conveniently daily ingested as source of complex carbohydrates, the caryopses of colored rice, which are available all the year round and have no contraindications, are interesting functional foods for their content of anthocyanins and other phenolic compounds [14]. On the contrary, the consumption of red wines, fruits, and vegetables, which are generally considered the major sources of dietetic anthocyanins, is restricted either by the increasing alarm about the negative health effects of wines and ethanol [15] or by the short seasonal availability of violet fresh fruits and vegetables, such as grapes, blueberries, and eggplants. 

As far as the extraction of phenolics is concerned, an innovative direct Ultrasonic-Assisted Extraction (UAE) is proposed in this study. Thanks to the energy released in the cavitation phenomena caused by the ultrasonic waves, UAE is particularly useful for the extraction of bioactive compounds from vegetable sources, since it reduces the solvent volumes, the extraction time, and the consumed energies of the traditional extraction methods [16]. In 2011 the indirect UAE extraction using an ultrasonic bath had been already proposed for the extraction of antioxidant compounds from rice husks [17]. However, the ultrasonic baths are less powerful then the ultrasonic probes employed for the proposed direct pulsed UAE, since the probes deliver higher ultrasonic intensity by direct immersion into the homogenized samples, thus enhancing the extraction yields. 

Starting from these observations, the steps of this study included:

- developing an efficient and environmental friendly method for the extraction of anthocyanins and other phenolic compounds from the caryopses, flour, and leaves of ‘Violet Nori’;
- evaluating the antioxidant properties and the anthocyanin content of the extracted samples and their changes in leaves throughout plant growth;
- singling out the best and most economically handled raw material for the sustainable industrial extraction of its anthocyanins.

The final aim was to investigate the healthy properties of the caryopses of this new violet cultivar and to single out the best economic source for the industrial extraction of anthocyanin among the different parts of the plant.

## 2. Results 

### 2.1. Preliminary Experiments

The intense violet color of both caryopses and leaves of ‘Violet Nori’ encouraged the preliminary attempts to extract their phenolic antioxidants. In this explorative phase, a conventional three-step extraction with acidified methanol and acetone [18] was performed on 1 g of powdered whole, brown, and parboiled ‘Violet Nori’ caryopses and husks, as well as on 1 g of its fresh leaves, cut into pieces about 5 mm in length. The Radical Scavenging Activities (RSAs) and the Total Phenolic Contents (TPCs) were determined in all of the extracted samples.

The values of RSA obtained from violet leaves were approximately 2-fold higher than those obtained from the corresponding whole caryopses, thus supporting the hypothesis that leaves are a possible raw material for the industrial extraction of substances with strong anti-radical activity. On the contrary, the preliminary trials on chaffs and husks did not give the hoped results: the TPCs measured by the Folin-Ciocalteu method [19] stood for the presence of phenolic compounds, but their RSAs [20] were so low that the results discouraged further investigation on these byproducts of rice production. Moreover, in these trials the RSAs of whole and brown rice were comparable, whereas the lower results of both RSAs and TPCs obtained for parboiled rice confirmed the assumption that the parboiling process depletes the amounts of valuable molecules. 

### 2.2. The First Tentative Direct UAE

**Figure 1:**
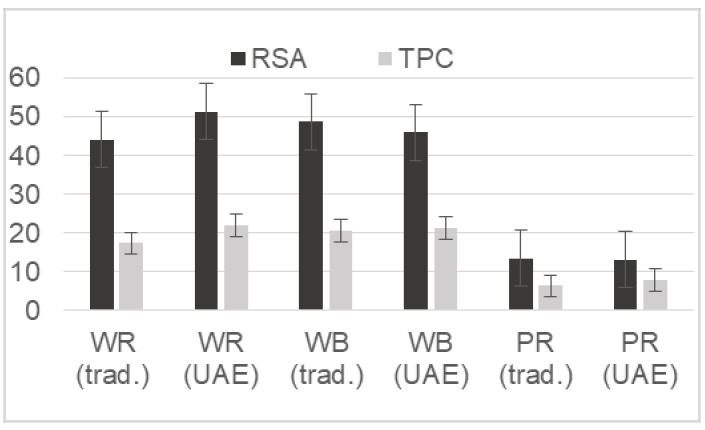
The Radical Scavenging Activity (RSA) and the Total Phenolic Content (TPC) obtained from ‘Violet Nori’ whole (WR), brown (BR), and parboiled (PR) rice grains by the traditional three-step extraction (trad.) and by the innovative direct Ultrasonic-Assisted Extraction (UAE).

The encouraging preliminary tests led to the tentative exploration of the employment of direct UAE for a more exhaustive extraction from both leaves and caryopses of ‘Violet Nori’, in sight of a possible extraction on an industrial scale. Ethanol 60% (pH = 5.8) was employed for UAE since, unlike methanol, it is considered eco-friendly and non-toxic for the extraction of bioactive compounds from plants [21], and it is GRAS (Generally Recognized as Safe) according to the Food and Drug Administration [22]. The 60% composition of the ethanolic solution was singled out as a compromise between the optimal 65–67% ethanol composition previously reported by Tabaraki and Nateghi [23], with the aim of reducing solvent consumption and costs. The extraction yields obtained by applying pulsed direct UAE to the whole, brown, and parboiled violet caryopses were evaluated by the RSAs and TPCs of the extracted solution and compared with the yields obtained by the three-step method [18] on the same samples. Figure 1 shows that the yields of the two methods were comparable (*p* < 0.01), even if the UAE conditions had been set rather arbitrarily and should be still improved. On the other hand, the employment of UAE significantly reduced both the time of extraction (about 15 min vs. 4 h) and the solvent volume (24 mL vs. 45 mL/g rice). Thus, to enhance the extraction yields, UAE conditions were improved by Design of Experiment (DoE), as reported in Section 4.4. 

### 2.3. The Selected UAE Conditions

The application of DoE allowed us to determine that only two UAE variables, i.e. pulse and sample/solvent ratio, were significant to the yields of extraction. Thus, these variables were optimized by Response Surface Methodology. Nevertheless, in order to reduce solvent volumes and costs, their values set for further experiments was a bit different from the optimized ones (see Section 4.4), since a ratio of 1:40 g/mL and a pulse of 80% fulfilled the aims of both obtaining a high extraction yield and limiting solvent volumes. The amplitude was set at 30%, since a further increase of the intensity of vibration proved to be useless. The final conditions are reported in Section 4.5.

### 2.4. Analyses of the Caryopses and Flour of ‘Violet Nori’ and Comparison with the Caryopses of Other Black Cultivars

In the following year’s crop, the improved UAE conditions were employed to extract total phenols and radical scavengers from the brown caryopses, flour, and leaves of ‘Violet Nori’ plants as well as from the caryopses of some prevalent black rice cultivars (‘Artemide’, ’Nero’, ‘Nerone’, ‘Otello’, and ‘Venere’). 

In this stage of the study two further analytical determinations were employed: the Oxygen Radical Absorbing Capacity (ORAC) method [24,25] was used since it has a radical quenching mechanism completely based on Hydrogen Atom Transfer (HAT), and its association with DPPH-test allows a better evaluation of compounds having different antioxidant mechanisms [26]. Moreover, HPLC-DAD was employed for the determination of anthocyanins in the solutions obtained by UAE from the different cultivars, since their color made them particularly interesting, even as possible natural colorants in food. The HPLC analysis was carried out by directly injecting the ethanol/water solutions obtained by the fast direct UAE of the caryopses and flour, since the results proved to be not significantly different (*p* < 0.01) from those obtained by an extraction method specific for rice anthocyanins [13], but involved 6 h of soaking in 80% methanol. In order to preserve anthocyanin integrity, the UAE solutions were stored at T = −20 °C until the time of analysis. In these conditions their content was stationary up to at least three months.

**Figure 2:**
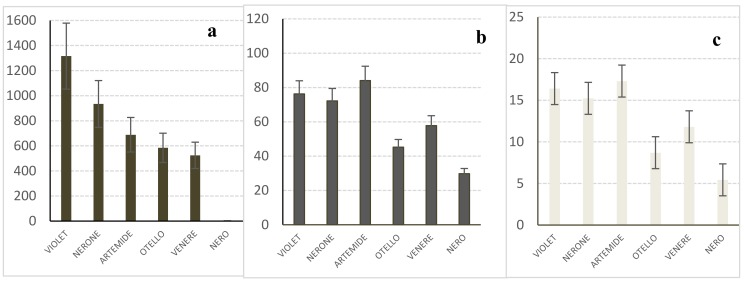
The results obtained from the analyses of the caryopses of different cultivars. (**a**) Anthocyanin content (μg/g, as cyanidin-3 glucoside); (**b**) RSA (μmol TEAC/g; TEAC = Trolox Equivalent Antioxidant Capacity); (**c**) TPC (mg GAE/g; GAE = Gallic Acid Equivalent).

Cyanidin-3 glucoside was by far the major detected anthocyanin both in ‘Violet Nori’ and in the other cultivars investigated in this study, along with lower detected amounts of peonidin-3-glucoside and cyanidin-3-rutinoside. As far as their total amounts are concerned, the comparison among the brown caryopses of ‘Violet Nori’ and those of the other black rice cultivars (Figure 2a) showed that the total anthocyanin content of ‘Violet Nori’ was generally higher than that of the other analyzed cultivars. Nevertheless, *Oryza sativa* L. ‘Artemide’ and ‘Nerone’ had RSA (Figure 2b) and TPC (Figure 2c) values comparable to those of ’Violet Nori’ and ‘Venere’, which is by far the most common colored rice in Italy, and had RSA values that were only slightly lower than that of ‘Violet Nori’.

The results obtained from the analysis of ‘Violet Nori’ caryopses and flour showed a close correlation between their TPCs and anthocyanin contents (R^2^ = 0.9672) and between RSAs and TPCs (R^2^ = 0.8839). 

### 2.5. Analyses of the Leaves of ‘Violet Nori’

**Figure 3:**
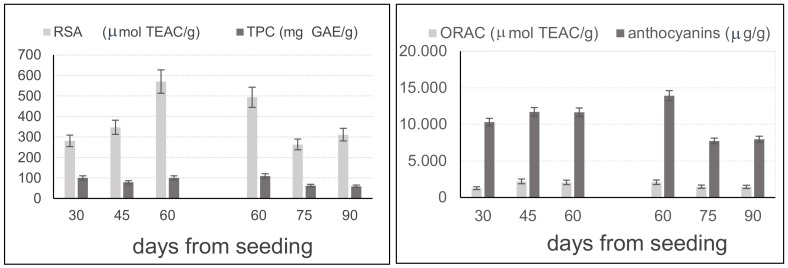
Changes of the measured analytical parameters in ‘Violet Nori’ leaves during the vegetative plant life. Results are expressed on a dry basis.

As far as leaves are concerned, the study was continued with the aim of both evaluating their possible employment as a raw material for the extraction of valuable antioxidants and singling out the best time interval for their cut. The leaves were cut at a height of about 10 cm in 1 m^2^ areas at 15-day intervals, i.e. at different plant maturation stages. Two sets of leaves were collected from two neighboring fields: the former in the second months from its seeding at 15-day intervals, i.e. after 30, 45, and 60 days, and the latter in the third month, i.e. after 60, 75, and 90 days from its seeding. This allowed an evaluation of the trend of the content of phenolic compounds and of their antioxidant activity after leaves had reached a height of at least 20 cm and up to 50–70 cm after 3 months from seeding, when leaves collection should not still threaten the further development of the ears. In this period, a parallel slight increase of leaves width from 3–4 mm to 6–7 mm was observed. Since the water contents detected in leaves appeared to be rather variable (between 60 and 70%) and severely influenced by climatic conditions, the results obtained for leaves extracts were expressed on a dry basis (Figure 3). 

Within each set of leaves, a high correlation (R^2^ > 0.988) was found between the ORAC values and the anthocyanin content, whereas the correlation between ORAC and RSA values was high (R^2^ = 0.9556) only between 60 and 90 days. Considering the two sets of samples, Figure 2 shows that RSA and ORAC values enhanced up to about 60 days from seeding, and then significantly decreased in the following month. A similar trend is visualized for anthocyanins, whereas the TPC appears rather constant up to 60 days and then decreases when leaves lose their firmness and elasticity.

In order to evaluate the stability of antioxidants from leaves under refrigerated conditions, the leaves collected at 30 and 60 days after seeding were also analyzed after 15 and 30 days of refrigeration at a temperature below 4 °C. A significant decrease of both RSAs and TPCs was already observed after 15 days of storage, when leaves appeared flabby and less elastic, and a further decrease was observed after 30 days, when bad odors started to develop. 

**Figure 4:**
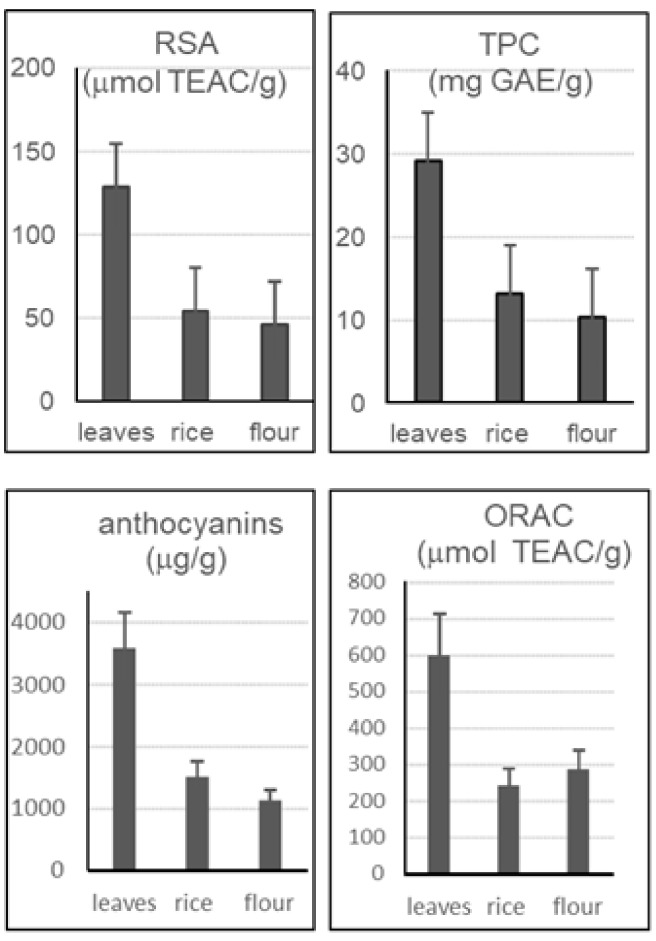
Comparison between ‘Violet Nori’ carypopses, flour, and leaves from the same year crop. Results are expressed on a ‘tel quel’ basis.

Finally, Figure 4 compares the medium values of the analytical results (expressed on a ‘tel quel’ weight) obtained from fresh leaves, caryopses, and packed industrial flours of ‘Violet Nori’ obtained in the same year crop. The results obtained from leaves were from 2–3 times higher than that obtained in rice and flours, and confirmed that leaves could be a very interesting raw material for the extraction of valuable antioxidants, particularly anthocyanins. 

## 3. Discussion

After the development of the environmental friendly direct UAE method, the results obtained from the analysis of the caryopses confirmed the previously reported antioxidant properties of the most common black rice varieties grown in Italy [14]. Moreover, by comparing the results obtained in the same UAE conditions from ‘Violet Nori’ caryopses and from the other analyzed black rice cultivars, the anthocyanins content of ‘Violet Nori’ rice appears outstanding. The high correlation between ORAC and RSA values (R^2^ = 0.9647), and between anthocyanin content and ORAC and RSA values (R^2^ = 0.8719 and R^2^ = 0.8668, respectively) of violet rice samples confirmed the significant activity of this class of flavonoids when oxidation involves a radical mechanism. The detected anthocyanin and antioxidant contents in its caryopses confirmed the great interest of ‘Violet Nori’ as a dietetic source of valuable health-promoting compounds.

The analyses of ‘Violet Nori’ flour largely confirmed the results obtained for the caryopses, although flour is generally obtained from grains discarded for their size or their blemishes. In fact, flour largely maintains the treasure of antioxidant molecules. This result is particularly important since this particular byproduct of rice processing could be employed not just for the extraction of valuable antioxidants but also as an ingredient for the production of valuable food products, for example for celiac disease. 

As far as leaves are concerned, the experiments during the vegetative plant cycle show that the leaves contents of TPCs and anthocyanins are 2–3-fold higher than those in rice and flour and reach their maxima at about 60 days from seeding. Thus, a predictable yield of about 4 kg anthocyanin/t fresh leaves is significantly higher than those of 1 kg anthocyanin/t rice, calculated on the basis of the medium anthocyanin amounts detected in ‘Violet Nori’ rice (1300 μg/g rice, as cyanidin-3-glucoside) for a yield of about 68 kg of rice from 100 kg paddy. However, the results obtained after leaves refrigeration demonstrate that fresh leaves should be extracted as soon as possible after their collection in order to prevent the deterioration of the active molecules. These interesting results will direct further experiments aiming both at evaluating the feasibility and the advantage of UAE for the extraction of anthocyanins from leaves on an industrial scale and at verifying whether leaves collection at this maturation stage really allows the further full development of the rice ears and the maturation of their caryopses. 

## 4. Materials and Methods

### 4.1. Chemicals

NaH_2_PO_4_•H_2_O (Monosodium phosphate monohydrate), Na_2_HPO_4_•7H_2_O (Disodium phosphate heptahydrate), DPPH• (1,1-diphenyl-2-picrylhydrazyl), Folin Ciocalteu reagents, Gallic acid, Trolox (6-hydroxy-2,5,7,8-tertramethylchromane-2 carboxylic acid), di-sodium fluorescein (FL), cyanidine-3-*O*-glucoside chloride, AAPH [2,2′-Azobis(2-amidinopropane) dihydrochloride], and ethanol 96° were supplied by SIGMA Aldrich (Milan, Italy). 

### 4.2. Equipment

An IKA Ultra-Turrax T25 (Staufen im Breisgau, Germany) realized a coarse homogenization of the samples in the extraction solvents. An Hielscher UP200St (Teltow, Germany) was employed for the direct UAE. An UV-Vis Agilent 8453 (Waldbronn, Germany) allowed the determinations of the TPC and RSA, whereas a 55LS Perkin Elmer Fluorescence Spectrometer (Waltham, MA, USA) was employed for the Oxygen Radical Absorbing Capacity (ORAC) determination.

### 4.3. Plant Materials 

‘Violet Nori’ rice samples were supplied by Azienda Agricola Eleonora Bertolone (Collobiano, VC, Italy) together with its leaves, flour, husks, and chaffs. They were collected in 2014–2017 in the area between Collobiano (VC) and Quinto Vercellese (VC), Italy, using the standard farming practices of that geographical area. The samples of black caryopses of the other analyzed cultivars were purchased from local markets. The leaves, from 20 to 60 cm long, were analyzed one day after their collection, and they were preliminary cut by hand into pieces of about 0.5 cm in length for the analyses.

### 4.4. Experimental Design for Improving UAE Yields

The experimental conditions of UAE were progressively improved by applying DoE, performed by an R-based chemometric software [27]. At first a Full Factorial Design was employed to investigate the influence of four quantitative UAE variables (instrumental amplitude (X_1_), pulse (X_2_), and time (X_3_) + sample/solvent ratio (X_4_)) on the extraction yields. Each variable was tested at two levels (Table 1). For a better evaluation of the extraction yields, two response variables were employed (Y_1_ = RSA (μmol TEAC/g sample), and Y_2_ = TPC (mg GAE/g sample)).

The sixteen experiments plus two replicates of the central point were carried out in a randomized order and allowed the estimation of the coefficients of the following resulting models:

Y_1_ = 43.55 + 3.45X_1_ + 4.37X_2_ + 1.65X_3_ − 4.30X_4_ 0.01X_1_X_2_ − 1.97X_1_X_3_ − 1.47X_1_X_4_ − 0.42X_2_X_3_ − 0.51X_2_X_4_ + 1.96X_3_X_4_ (1)

Y_2_ = 11.23 + 0.73X_1_ + 1.3341X_2_ + 0.43X_3_ − 0.47X_4_ − 0.13X1X _2_− 0.51X_1_X_3_ + 0.19X_1_X_4_ + 0.12X_2_X_3_ − 0.23X_2_X_4_ − 0.28X_3_X_4_ (2)

**Figure 5:**
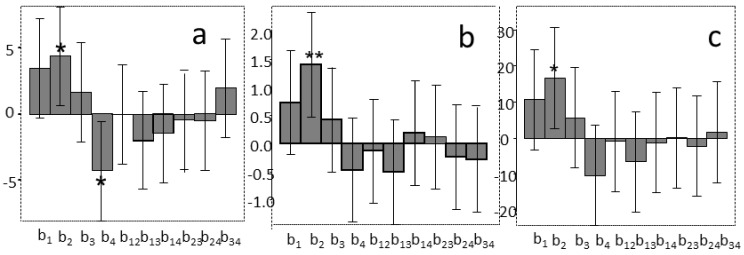
The coefficients of the models of (**a**) Y_1_ (μmol TEAC/g sample), (**b**) Y_2_ (mg GAE/g sample), and (**c**) Y_3_ (medium value of the corresponding scaled Y_1_ and Y_2_) obtained by the Full Factorial Design (X_1_, amplitude; X_2_, pulse; X_3_, time; X_4_, sample/solvent ratio).* = *p* < 0.05, ** = *p* < 0.01.

Looking at the plots of their coefficients (Figure 5a,b), both pulse (X_2_), and sample/solvent ratio (X_4_) were shown to be significant (*p* < 0.05) for Y_1_, whereas Y_2_ was affected only by pulse (X_2_), but at a higher level of significance (*p* < 0.01). In both models in the studied interval when the higher was pulse, the estimated yields were higher. However, the influence of X_2_ and X_4_ should be further studied in order to improve the extraction yields. 

Before proceeding, the amplitude significance was further tested, since the error bar of X_1_ was just a little lower than zero, which means that amplitude was also nearly significant. The values of Y_1_ and Y_2_ of each experiment were thus scaled to obtain a new response variable Y_3_ by the medium value of the scaled Y_1_ and Y_2_. The Y_3_ model, visualized in Figure 5c, confirmed that amplitude was not significant for the extraction yield.

Y_3_ = 56.10 + 10.69X_1_ + 16.65X_2_ + 5.67X_3_ − 10.43X_4_ − 0.88X_1_X_2_ − 6.70X_1_X_3_ − 1.317X_1_X_4_ + 0.04X_2_X_3_ − 2.38X_2_X_4_ + 1.58X_3_X_4_ (3)

**Figure 6:**
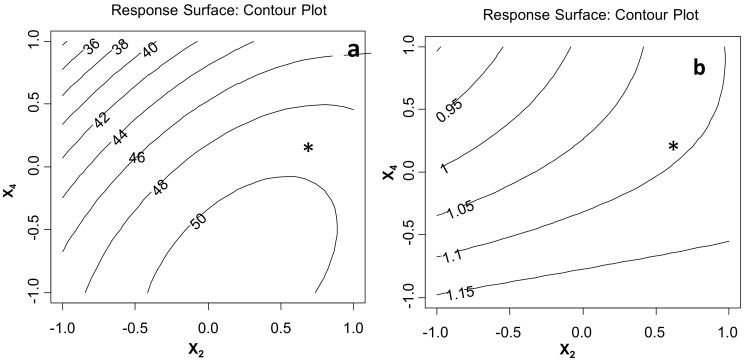
The Response Surface Contour Plots obtained for (**a**) Y_1_ and (**b**) Y_2_ and the mathematical models obtained by the Central Composite Design (X_2_, pulse; X_4_, sample/solvent ratio). * locates the selected conditions.

Then, a faced Central Composite Design was applied to the two selected significant variables, X_2_ and X_4_, in order to study their interaction and quadratic terms. The variable levels were modified according to the results of the factorial design: X_2_ was increased and studied between 50% (+1) and 90% (−1), since it had a positive effect on both Y_1_ and Y_2_, and X_4_ was decreased between 1:30 (+1) and 1:60 (−1), since it had a negative effect on Y_1_. The obtained models and the contour plots of the response surfaces of Y_1_ and Y_2_ are reported in Figure 6, confirming that Y_1_ and Y_2_ were higher at higher sample dilutions (X_4_). On the contrary, neither X_2_ nor the quadratic terms nor the interaction were significant at these levels. The Y_1_ plot visualizes that the higher values of TEAC could be obtained with X_4_ between 1:45 and 1:60 g/mL at a pulse between approximately 60 and 85%. Nevertheless, in sight of reducing solvent costs and environmental impact, a ratio of 1:40 g/mL was preferred, since it led to responses that were not much lower than the best values. Furthermore, looking at the plot in Figure 6b, an 80% pulse value, corresponding to an 80% compression phase, was then selected, since higher values of the high-pressure phase could prevent the formation of cavitation bubbles in the residual low-pressure phase [28]. The projection of X_2_ = 80% and X_4_ = 1:40 on Y_1_ and Y_2_ plots located two points, indicated as *, close to iso-contours Y_1_ = 49 and Y_2_ = 1.10, respectively, i.e. not far from the Y_1_ = 50 and Y_2_ = 1.15 values that could be obtained with a higher solvent amount. These X_2_ and X_4_ values were chosen for further experiments.

### 4.5. The Final UAE Conditions

The final UAE conditions were as follows: 40 mL of ethanol 60% were added to 1 g of powdered rice (or husks or flour or 0.5 cm pieces of leaves) in a polypropylene 50 mL conical centrifuge tube, which was then immersed in an ice-bath for a temperature-controlled homogenization. The sample was homogenized by the Ultra-Turrax T25 for 2 min (0.5 min at 8000 rpm + 1.5 min at 24,000 rpm) before sonication. Then, a 7 mm e.d. (external diameter) sonotrode was immersed into the tube for direct sonication for 5 min, at 30% amplitude and 80% pulse parameters. During the UAE process, the temperature never exceeded 60 °C and a further homogenization was realized. The obtained suspension was filtered by a Whatmann n. 1 paper disk and a further filtration by an RC 0.45 μm membrane syringe filter was required for the instrumental analyses of the solutions.

### 4.6. Determination of TPC

TPC was determined for all of the solutions extracted in this study, whether obtained by traditional or UAE extraction. TPC was determined by the Folin Ciocalteu method [19]. A multi-level calibration at λ = 760 nm with gallic acid allowed expressing results as mg GAE/g sample (GAE = Gallic Acid Equivalent). Different dilutions of the extracted solution were appropriate, i.e. 1:10 for the solution obtained from rice caryopses, husks, and flour, and 1:25 for the solutions obtained from leaves. 

### 4.7. Determination of RSA

RSA was determined for all of the solutions extracted in this study, whether obtained by traditional or UAE extraction. RSA was determined with DPPH• [20]. A multi-level calibration with Trolox at λ = 515 nm allowed expressing the results as μmol TEAC/g sample (TEAC = Trolox Equivalent Antioxidant Capacity). No dilution was necessary for the solutions obtained from the caryopses, husks, and flour, whereas a 1:10 dilution was appropriate for the solutions obtained from leaves. 

### 4.8. Determination of the Oxygen Radical Absorbing Capacity (ORAC) 

ORAC was measured in the solutions obtained by applying the final UAE conditions to ‘Violet Nori’ brown caryopses, flour, and leaves. A Perkin Elmer 55LS was equipped with a single-cell holder so that, starting from the conditions reported for the microplate assay [24] and the information retrieved for the single-cell determination [26], the method was adapted for single-cell employment. The cell temperature was carefully set at 37 ± 1 °C. A 0.75 mM phosphate buffer at pH = 7.4 was employed for the preparation of the solutions and for the sample dilution. Three milliliters of FL 6.64 × 10^−5^ mM was accurately pre-incubated at 37 ± 1°C for exactly 15 min before adding 50 μL of sample and 50 μL of AAPH 0.6 M in rapid sequence. The obtained solution was immediately vortexed for 10 s, transferred into a 1 cm quartz vial, and introduced into the single-cell holder, so that the first fluorescence reading (f_0_) was exactly 30 s after the AAPH addition. The fluorescence intensity was measured at λex = 490 and λem = 515 nm every minute up to 30 min (f_n_,_30_), when the fluorescence decay was practically completed (residual fluorescence was lower than 2% of f_0_). Instrumental parameters were set up in order to obtain each f value as the average of three consecutive readings. 

For each standard solution or sample, the Area Under the fluorescence decay Curves (AUC) and the net relative AUC, i.e. the value obtained by subtracting the AUC of the blank (the phosphate buffer) from the AUC of the standard or sample, were obtained [25]. The ORAC values were determined from the Trolox calibration curve and expressed as μmol TEAC/g sample (R^2^ = 0.99). The Trolox calibration curve was daily repeated with four Trolox standard solutions at concentrations between 12.5 and 100 μM.

During the development of this method, a careful control and standardization of temperatures, times, and sequence of reagent additions proved to be critical for the method repeatability.

### 4.9. HPLC Determination of the Anthocyanin Content

A reversed phase HPLC method reported for phenolic compounds in bud extracts [29] was slightly modified and then employed for the determination of the anthocyanin content in the solutions obtained by applying the improved UAE conditions to ‘Violet Nori’ brown caryopses, flour, and leaves and to the solutions obtained by UAE from the caryopses of the other cultivars. A C-18 core-shell column (Agilent Poroshell 120 EC, 3 mm × 150 mm, 2.7 μm particle size) was employed, with a mobile phase of water acidified at pH 2.14 by formic acid (solvent A) and acetonitrile (solvent B), with a 0.4 mL/min flow rate, and a 30 °C column temperature. The multi-step gradient was 95% A at 0 min, 70% A at 25 min, 45% A at 35 min, and 0% A at 42 min, held for 3 min. Anthocyanins were detected and quantified at 515 nm by external standard quantification with a four-point cyanidin-3 glucoside regression line (R^2^ = 0.9998). Results were expressed as μg cyanidin-3-glucoside/g of raw material. 

## Figures and Tables

**Table 1:** The experimental plan of the Factorial Design and the obtained responses.

Experiment	X_1_^a^	X_2_	X_3_	X_4_	Y_1_	Y_2_
						
1	30%	20%	5 min	1:40	35.8	7.4
2	70%	20%	5 min	1:40	52.3	12.7
3	30%	80%	5 min	1:40	47.7	12.0
4	70%	80%	5 min	1:40	54.5	11.7
5	30%	20%	15 min	1:40	33.1	10.0
6	70%	20%	15 min	1:40	48.2	10.0
7	30%	80%	15 min	1:40	52.7	15.1
8	70%	80%	15 min	1:40	53.7	14.4
9	30%	20%	5 min	1:32	25.5	8.1
10	70%	20%	5 min	1:32	32.5	9.7
11	30%	80%	5 min	1:32	34.5	10.6
12	70%	80%	5 min	1:32	47.7	13.9
13	30%	20%	15 min	1:32	46.0	10.2
14	70%	20%	15 min	1:32	35.2	10.2
15	30%	80%	15 min	1:32	40.7	10.3
16	70%	80%	15 min	1:32	47.2	12.8
17	50%	50%	10 min	1:36	47.1	11.2
18	50%	50%	10 min	1:36	49.5	11.9

^a^ X_1_, amplitude; X_2_, pulse; X_3_, time; X_4_, sample/solvent ratio; Y_1_, RSA (μmol TEAC/g sample); Y_2_, TPC (mg GAE/g sample).

## References

[1] Italian Ministry of agricultural food and forestry policies. http://www.enterisi.it/servizi/Menu/dinamica.aspx?idSezione=17505&idArea=17548&idCat=17552&ID=17552&TipoElemento=categoria.

[2] Kim M.-K., Koh H.-A., Koh K., Kim H.-S., Lee Y.-S., Kim Y.-H. (2008). Identification and quantification of anthocyanin pigments in colored rice. Nutr. Res. Pract..

[3] Mazza G., Gao L., Abdel-Aal E., Wood P. (2005). Blue and purple grains. Specialty Grains for Food and Feed.

[4] Goufo P., Trindade H. (2014). Rice antioxidants: phenolic acids, flavonoids, anthocyanins, proanthocyanidins, tocopherols, tocotrienols, γ-oryzanol, and phytic acid. Food Sci. & Nutr..

[5] Abdel Aal E.S.M., Young J.C., Rabalski I. (2006). Anthocyanin composition in black, blue, pink, purple, and red cereal grains. J. Agric. Food Chem..

[6] Chiang A.N., Wu H.L., Yeh H.I., Chu C.S., Lin H.C., Lee W.C. (2006). Antioxidant effects of black rice extract through the induction of superoxide dismutase and catalase activities. Lipids.

[7] Nam S.H., Choi S.P., Kang M.Y., Koh H.J., Kozukue N., Friedman M. (2006). Antioxidative activities of bran from twenty-one pigmented rice cultivars. Food Chem..

[8] Zhang M.W., Zhang R.F., Guo B.J., Chi J.W., Wei Z.C., Xu Z.H. (2006). The hypolipidemic and antioxidative effects of black rice pericarp anthocyanin in rats. Acta Nutr. Sinica.

[9] Shen Y., Jin L., Xiao P., Lu Y., Bao J.S. (2009). Total phenolics, flavonoids, antioxidant capacity in rice grain and their relations to grain color, size and weight. J. of Cereal Sci..

[10] Hyun J.W., Chung H.S. (2004). Cyanidin and malvidin from *Oryza sativa* cv. Heugjinjubyeo mediate cytotoxicity against human monocytes leukemia cells by arrest of G 2 /M phase and induction of apoptosis. J. Agric. Food Chem..

[11] Zhao C., Giusti M.M., Malik M., Moyer M.P., Magnuson B.A. (2004). Effects of commercial anthocyanin-rich extracts on colonic cancer and nontumorigenic colonic cell growth. J. Agric. Food Chem..

[12] Tsuda T., Horio F., Osawa T. (2002). Cyanidin 3-*O*-β-glucoside suppresses nitric oxide production during a zymosan treatment in rats. J. Nutr. Sci. Vitamin..

[13] Chen X.Q., Nagao N., Itani T., Irifune K. (2012). Anti-oxidative analysis and identification and quantification of anthocyanin pigments in different coloured rice. Food Chem..

[14] Bordiga M., Gomez Alonso A., Locatelli M., Travaglia F., Coïsson J.D., Hermosin Gutierrez I., Arlorio M. (2014). Phenolics characterization and antioxidant activity of six different *Oryza sativa* cultivars grown in Piedmont (Italy). Food Res. Int..

[15] International Agency for Research on Cancer (IARC) (2012). Monograph.

[16] Azmir J., Zaidul I.S.M., Rahman M.M., Sharif K.M., Mohamed A., Sahena F., Jahurul M.H.A., Ghafoor K., Norulaini N.A.N., Omar A.K.M. (2013). Techniques for extraction of bioactive compounds from plant materials: A review. J. Food Eng..

[17] Ghasemzadeh A., Jaafar H.Z.E., Juraimi A.S., Tayeb Meigooni A. (2015). Comparative Evaluation of Different Extraction Techniques and Solvents for the Assay of Phytochemicals and Antioxidant Activity of Hashemi Rice Bran. Molecules.

[18] Walter M., Marchesan E., Sachet Massoni P.F., Picolli da Silva L., Meneghetti Sarzi Sartori G., Bruck Ferreira R. (2013). Antioxidant properties of rice grains with light brown, red and black pericarp colors and the effect of processing. Food Res. Int..

[19] Singleton V.L., Orthofer R., Lamuela-Raventos R.M. (1999). Analysis of total phenols and other oxidation substrates and antioxidants by means of Folin-Ciocalteu reagent. Meth. Enzym..

[20] Brand-Williams W., Cuvelier M.E., Berset C. (1995). Use of a free radical method to evaluate antioxidant activity. LWT-Food Sci. Technol..

[21] Bartnik D.D., Mohler C.M., Houlihan M. (2006). Methods for the production of food grade extracts. U.S. Patent.

[22] U.S. Food and Drug Administration CFR—Code of Federal Regulations Title 21, B, 184, 2018. https://www.ecfr.gov/cgi-bin/text-idx?SID=08d7b882ac7656d40f2f422caee53d25&mc=true&node=pt21.3.184&rgn=div5.

[23] Tabaraki R., Nateghi A. (2011). Optimization of ultrasonic-assisted extraction of natural antioxidants from rice bran using response surface methodology. Ult. Sonochem..

[24] Cao G.H., Prior R.L. (1999). Measurement of oxygen radical absorbance capacity in biological samples. Oxidants and anti-oxidants. Meth. Enzym..

[25] Ou B., Hampsch-Woodill M., Prior R.L. (2001). Development and validation of an improved oxygen radical oxygen assay using fluorescein as the fluorescent probe. J. Agric. Food Chem..

[26] Schaich K.M., Tian X., Xie J. (2015). Reprint of “Hurdles and pitfalls in measuring antioxidant efficacy: A critical evaluation of ABTS, DPPH, and ORAC assays”. J. Funct. Food.

[27] Italian Chemical Society Division of Analytical Chemistry-Group of Chemometrics. R-Based Chemometric Software. http://gruppochemiometria.it/index.php/software/.

[28] Santos H.M., Lodeiro C., Capelo-Martínez J.L. (2009). The Power of Ultrasound. Ultrasound in Chemistry: Analytical Applications.

[29] Ieri F., Innocenti M., Possieri L., Gallori S., Mulinacci N. (2015). Phenolic composition of “bud extracts” of *Ribes nigrum* L., *Rosa canina* L., and *Tilia Tomentosa* M. J. Pharm. Biomedic. Anal..

